# Antibiotic resistance in ocular bacterial infections: an integrative review of ophthalmic chloramphenicol

**DOI:** 10.1186/s41182-023-00496-x

**Published:** 2023-03-09

**Authors:** Babatunde Ismail Bale, Emmanuel Ebuka Elebesunu, Pirakalai Manikavasagar, Favour Obianuju Agwuna, Isaac Olushola Ogunkola, Alhaji Umar Sow, Don Eliseo Lucero-Prisno

**Affiliations:** 1 Department of Optometry, University of Benin, Edo, Nigeria; 2 Department of Medical Laboratory Sciences, University of Nigeria, Enugu, Nigeria; 3 Public Health for Eye Care, London School of Hygiene and Tropical Medicine, London, UK; 4 Faculty of Pharmaceutical Sciences, University of Nigeria, Enugu, Nigeria; 5 Department of Public Health, University of Calabar, Calabar, Nigeria; 6 College of Medicine and Allied Health Sciences, University of Sierra Leone, Freetown, Sierra Leone; 7 Department of Global Health and Development, London School of Hygiene and Tropical Medicine, London, UK

**Keywords:** Chloramphenicol, Drug, Antibiotic resistance, Ophthalmic, Infection, Treatment

## Abstract

**Introduction:**

Chloramphenicol is a broad-spectrum antibiotic widely used for treating ophthalmic infections, but concerns about rising bacterial resistance to chloramphenicol have been observed due to its frequent use as an over-the-counter medication. This review assessed the common ophthalmic bacterial pathogens, their chloramphenicol resistance mechanisms, and rates of drug resistance.

**Methods:**

PubMed and Google Scholar databases were searched for relevant publications from the years 2000 to 2022, bordering on ophthalmic bacterial infections, chloramphenicol susceptibility profiles, and drug resistance mechanisms against chloramphenicol. A total of 53 journal publications met the inclusion criteria, with data on the antibiotic susceptibility profiles available in 44 of the reviewed studies, which were extracted and analyzed.

**Results:**

The mean resistance rates to chloramphenicol from antibiotic susceptibility profiles varied between 0% and 74.1%, with the majority of the studies (86.4%) showing chloramphenicol resistance rates below 50%, and more than half (23 out of 44) of the studies showed resistance rates lower than 20%. The majority of the publications (*n* = 27; 61.4%) were from developed nations, compared to developing nations (*n* = 14; 31.8%), while a fraction (*n* = 3; 6.8%) of the studies were regional cohort studies in Europe, with no country-specific drug resistance rates. No pattern of cumulative increase or decrease in ophthalmic bacterial resistance to chloramphenicol was observed.

**Conclusions:**

Chloramphenicol is still active against ophthalmic bacterial infections and is suitable as a topical antibiotic for ophthalmic infections. However, concerns remain about the drug becoming unsuitable in the long run due to some proof of high drug resistance rates.

## Background

Chloramphenicol is a common ophthalmic drug for treating bacterial eye infections in several countries worldwide. However, in the United States of America (USA) and certain other developed countries, chloramphenicol was banned due to its association with adverse effects, such as aplastic anaemia, acute leukaemia, contact dermatitis and anaphylaxis [1, 2]. It has also been associated with vision loss [3], and as of 2017, the European Medicines Agency (EMA) moved to prohibit the use of chloramphenicol eye drops in children under 2 years of age [4]. However, chloramphenicol is still very much in use in many developing countries, as it is cost-effective and easily accessible. The drug, originally called Chloromycetin, is a broad-spectrum antibiotic that was isolated from the soil bacterium *Streptomyces venezuelae* in 1947 and is known to function as an inhibitor of protein synthesis, possessing bacteriostatic action [5]. Chloramphenicol belongs to its own antibiotic class, along with its derivates, Florfenicol, Thiamphenicol and Azidamphenicol, which were synthesized by chemical substitutions with fluoro and sulfomethyl groups at specific positions of the primary chloramphenicol molecule [6]. The derivatives thiamphenicol and azidamphenicol are used alongside chloramphenicol in human medicine, while the fluorinated derivative florfenicol is used only in veterinary medicine. As a broad-spectrum antibiotic, chloramphenicol spectrum of activity includes Gram-positive, Gram-negative, anaerobic and aerobic bacteria, as well as other unconventional bacteria, such as mycoplasmas, rickettsia and chlamydia [6].

Chloramphenicol is available in eye drops for treating ophthalmic infections; however, some proof of drug resistance against chloramphenicol have been observed over the years, with some other antibiotics being considered as alternatives for more effective ophthalmic treatment. Ophthalmic chloramphenicol is useful as an over-the-counter medication, because it helps provide quick relief from the discomfort of ocular infections and also reduces the need for clinical consultations over mild ophthalmic infections. This was the rationale behind the decision by the Medicines and Healthcare Products Regulatory Agency (MHRA) of the United Kingdom (UK) to reclassify chloramphenicol eye drops, such that they could be more easily accessible in pharmacy outlets without needing a doctor’s prescription [7]. This move to increase access to safe and effective treatment with chloramphenicol led to a 15.5% fall in the number of doctors’ prescriptions of the drug, from 2.3 million in 2004 to 1.94 million in 2007, and was accompanied by a concomitant rise in over-the-counter pharmacist prescriptions of the drug from 0.68 million in 2005 to 1.46 million in 2007. This further led to a 47.8% increase in total chloramphenicol use between 2004 and 2007 [7]. As a result, the frequent and unregulated use of the drug has been said to be a driving factor for the emergence of chloramphenicol resistance, with growing opinions that there is a high rate of drug resistance to chloramphenicol in the treatment of both ophthalmic and systemic infections [8]. Chloramphenicol is known for its potent inhibitory effect on protein biosynthesis in bacteria, and due to its overuse in the treatment of ophthalmic infections, various mechanisms of resistance to the drug have emerged, either intrinsically developed or acquired through the dispersion of mobile genetic elements conveying chloramphenicol resistance genes, such as plasmids and transposons [9].

The objective of this review is to assess the bacterial pathogens commonly involved in ophthalmic infections, their resistance mechanisms against chloramphenicol, and the rates of drug resistance to chloramphenicol over the years. The research question is to ascertain if chloramphenicol resistance is highly prevalent or not.

## Methods

**Fig. 1:**
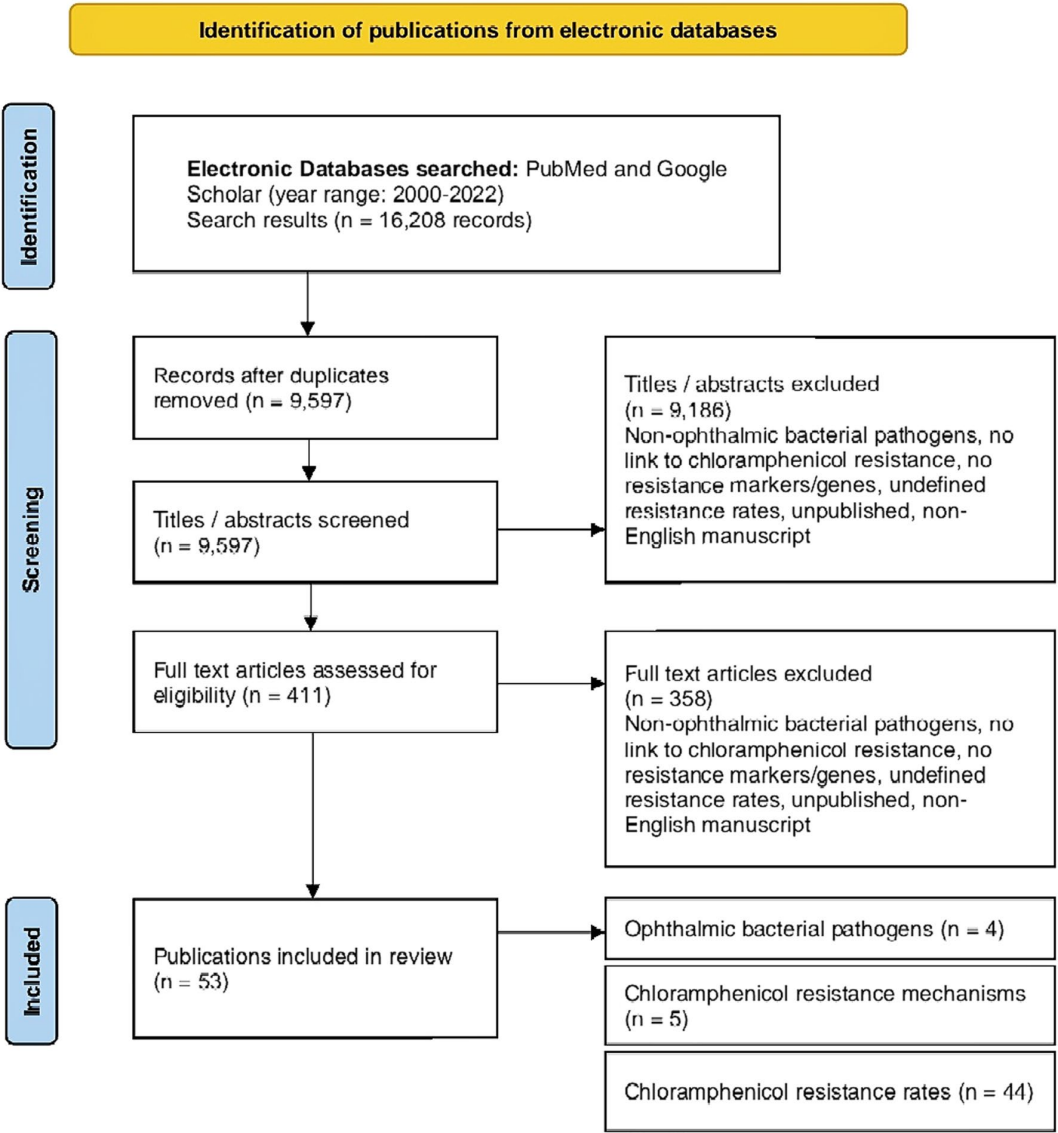
PRISMA flow diagram detailing the steps in obtaining literature for the review

PubMed and Google Scholar databases were searched for relevant publications bordering on ophthalmic bacterial infections, chloramphenicol use, and drug resistance mechanisms associated with chloramphenicol. The search terms used were; chloramphenicol resistance, ophthalmic infections, bacterial pathogens, antibiotic resistance, antibiotic susceptibility, eye treatment, drug efficacy, and drug resistance mechanisms. The inclusion criteria included accessible articles published from 2000 to 2022, related to ophthalmic infections in humans only, specific chloramphenicol resistance mechanisms, and involved chloramphenicol use for susceptibility tests or treatment. Only papers published in the English language were reviewed. Unpublished manuscripts were not included in the review. All publications falling short of these criteria were excluded. The review timeline of 2000–2022 was chosen, because most of the elaborate multicohort studies detailing chloramphenicol drug resistance rates were carried out within this time frame, as opposed to prior years when the topic of chloramphenicol resistance was not regularly explored. A total of 53 journal publications met the inclusion criteria, spanning the prevalent ophthalmic bacterial pathogens, resistance mechanisms, and resistance rates to chloramphenicol. Data on the antibiotic susceptibility and resistance profiles of ophthalmic bacterial pathogens to chloramphenicol were available in 44 of the reviewed studies, which were extracted, analyzed, and discussed to explore the aim of the study. A PRISMA flow diagram of the review and literature selection process is outlined in Fig. 1.

## Results

### Common bacterial pathogens involved in ophthalmic infections

A number of ocular bacterial infections exist, which include ophthalmic keratitis, conjunctivitis, blepharitis, endophthalmitis, dacryocystitis and orbital cellulitis [6]. It was observed from the reviewed publications that certain genera and species of bacteria were specifically associated with ophthalmic infections and drug resistance patterns. The types of bacterial ophthalmic infections and their commonly associated bacterial pathogens have been outlined in Table 1. Bacterial conjunctivitis was said to be the most prevalent ophthalmic infection, and *Staphylococcus *spp. was the most common causative bacterial agent in adults, while ophthalmic infections in children were more often caused by *Haemophilus influenzae* and *Streptococcus pneumoniae* [10, 11].

**Table 1:** Ophthalmic infections and their commonly associated bacterial pathogens

Ophthalmic infection	Associated bacterial pathogens	Category
Bacterial conjunctivitis	*Staphylococcus aureus*	Gram-positive
	*Staphylococcus epidermidis*	
	*Streptococcus pneumoniae*	
	Viridans streptococci group	
	*Haemophilus influenzae*	Gram-negative
	*Pseudomonas aeruginosa*	
	*Pseudomonas putida*	
	*Klebsiella pneumoniae*	
	*Escherichia coli*	
	*Enterococcus *spp*.*	
	*Proteus mirabilis*	
	*Neisseria meningitidis*	
	*Neisseria gonorrhoeae*	
	*Moraxella catarrhalis*	
	*Morganella morganii*	
	*Serratia marcescens*	
Bacterial keratitis	*Staphylococcus aureus*	Gram-positive
	*Staphylococcus epidermidis*	
	*Streptococcus pneumoniae*	
	*Bacillus *spp*.*	
	*Microbacterium liquefaciens*	
	*Haemophilus influenzae*	Gram-negative
	*Pseudomonas aeruginosa*	
	*Pseudomonas putida*	
	*Klebsiella pneumoniae*	
	*Serratia marcescens*	
	*Moraxella lacunata*	
Endophthalmitis	Coagulase-negative *Staphylococcus* (CoNS)	Gram-positive
	*Bacillus *spp*.*	
	*Streptococcus *spp*.*	
Blepharitis	*Staphylococcus aureus*	Gram-positive
	Coagulase-negative *Staphylococcus* (CoNS)	
Orbital cellulitis	*Staphylococcus aureus*	Gram-positive
	*Streptococcus pyogenes*	
	*Haemophilus influenzae*	Gram-negative
Dacryocystitis	*Pseudomonas aeruginosa*	Gram-negative
	*Escherichia coli*	
	*Enterobacter aerogenes*	
	*Citrobacter *spp*.*	
	*Enterococcus *spp*.*	
	*Staphylococcus aureus*	Gram-positive
	*Streptococcus pneumoniae*	

### Resistance mechanisms against chloramphenicol

A total of 37 chloramphenicol resistance markers were identified among the various ophthalmic bacterial pathogens, of which 23 (62.2%), 9 (24.3%), and 5 (13.5%) were borne on plasmids, transposons and chromosomes, respectively. Two types of chloramphenicol resistance mechanisms were prevalent, which included chloramphenicol acetyltransferase enzymes used to inactivate chloramphenicol by acetylation, and chloramphenicol efflux/export pumps used to actively expel the drug upon its entry into the bacterial cell. The identified genes encoding for chloramphenicol acetyltransferases (*cat*) were *cat I, II, B2–B8, P,* and *S*, while the genes encoding for chloramphenicol exporters (*cml*) were *cml A1, A4, A5, A6,* and *A7*. These resistance mechanisms were either intrinsically developed or acquired by the bacteria through the dispersion of mobile genetic elements conveying the said resistance genes. The details of the identified drug resistance mechanisms are outlined in Table 2.

**Table 2:** Drug resistance mechanisms involved in ophthalmic bacterial infections

Resistance mechanism	Resistance gene	Bacterial sources	Plasmid/Transposon/Chromosome
Chloramphenicol acetyltransferases	*cat (I, II, B2, B3, B4, B5, B6, B7, B8, P, S)*	*Escherichia coli*	Tn*9*
			R429 (*catI*)
			pSa (*catII*)
			pNR79:Tn2424 (*catB2*)
			pHSH2 (*catB3*)
		*Haemophilus influenzae*	pR1234
			pMR375 (*catII*)
		*Serratia marcescens*	R478
		*Proteus mirabilis*	Chromosome
		*Bacillus subtilis*	pTZ12
		*Staphylococcus aureus*	pKH7
			pUB112
			pC223
			pSCS6
			pSCS7
			pC194
			pMC524-MBM
		*Streptococcus pyogenes*	Chromosome (*catS*)
		*Klebsiella pneumoniae*	pEKP0787-1 (*catB4*)
			pKB42 (*catB8*)
		*Enterococcus *spp.	pRE25
			pRUM
		*Pseudomonas aeruginosa*	pPAM-101 (*catB6*)
			Chromosome (*catB7*)
			Plasmid (*catB8*)
		*Neisseria meningitidis*	Chromosome (*catP*)
		*Morganella morganii*	Tn*840* (*catB5*)
Chloramphenicol exporters	*cml (A1, A4 A5, A6, A7)*	*Escherichia coli*	R751 (Tn*2000*) (*cmlA5*)
			R26
		*Pseudomonas aeruginosa*	RPL11 (Tn*1403*) (*cmlA1*)
			pR1033:Tn*1696* (*cmlA1*)
			Plasmid (*cmlA6*)
			Chromosome (*cmlA7*)
		*Klebsiella pneumoniae*	pILT-3 (*cmlA1*)
			pTK1 (*cmlA4*)

### The rates of drug resistance to chloramphenicol

To assess drug resistance patterns over time, data on the mean susceptibility and resistance rates to chloramphenicol from antimicrobial susceptibility tests (ASTs) were drawn from the reviewed literature, along with information on the country or region and the year in which the studies were carried out or published. Varying rates of drug resistance were observed in various ophthalmic bacterial infections from the reviewed literature, which are outlined in Table 3.

**Table 3:** Mean susceptibility and resistance rates in ophthalmic bacterial infections from reviewed literature

Years	Country/Region	Mean susceptibility rate (%)	Mean resistance rate	References
2000	United Kingdom	67.9	32.1%	[12]
2000	Austria	85.7	14.3%	[13]
2001	Switzerland	82	18%	[14]
2002	Europe	85.9	14.1%	[15]
2002	Japan	81	19%	[16]
2002	India	75.2	24.8%	[17]
2003	United States of America	40	60%	[18]
2004	Brazil	90.6	9.4%	[19]
2004	United Kingdom	86.8	13.2%	[20]
2005	United Kingdom	100	N/A	[21]
2006	Brazil	92.1	7.9%	[22]
2007	Iran	66	34%	[23]
2008	Spain	95.2	4.8%	[24]
2008	Korea	66.7	33.3%	[25]
2009	Europe	78.3	21.7%	[26]
2009	Nigeria	69.2	30.8%	[27]
2010	United Kingdom	87.4	12.6%	[28]
2011	Nigeria	30.4	69.6%	[29]
2011	United States of America	97.3	2.7%	[30]
2011	United Kingdom	25.9	74.1%	[31]
2011	United Kingdom	65.9	34.1%	[32]
2012	Oman	63	37%	[33]
2013	Greece	97.6	2.4%	[34]
2013	Italy	75	25%	[35]
2014	Ethiopia	31.5	68.5%	[36]
2015	Bosnia and Herzegovina	99.4	0.6%	[37]
2015	Europe	85.9	14.1%	[38]
2016	Italy	74	26%	[39]
2016	China	92	8%	[40]
2016	United Kingdom	96.6	8.4%	[41]
2016	China	49	51%	[42]
2016	Australia	79	21%	[43]
2017	Ethiopia	87.5	12.5%	[44]
2017	Ethiopia	70.2	29.8%	[45]
2018	Japan	88	12%	[46]
2018	Ethiopia	73.8	26.2%	[47]
2018	United Kingdom	90	10%	[48]
2019	United States of America	97.8	2.2%	[49]
2019	United Kingdom	79.8	20.2%	[50]
2019	United States of America	96.3	3.7%	[51]
2019	India	95	5%	[52]
2020	Iran	30.2	69.8%	[53]
2021	Ireland	97.6	2.4%	[54]
2022	Ethiopia	69.9	30.1%	[55]

**Fig. 2:**
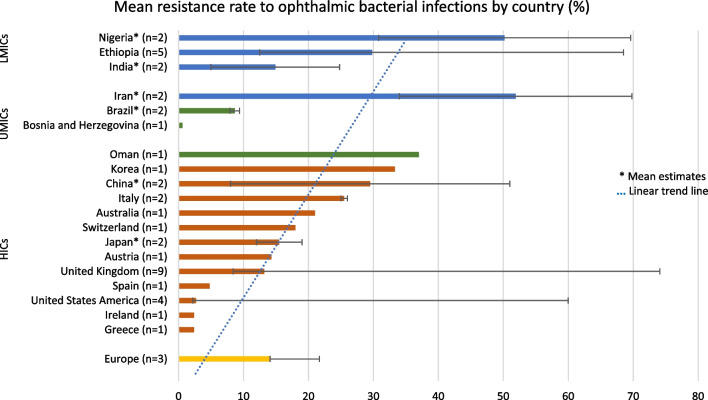
Graphical representation of the mean resistance rate to chloramphenicol by country/region

Based on the data from the above reviewed studies, a summary table (Table 4) and graph (Fig. 2) representing the drug resistance rates by country/region from the reviewed literature is depicted below.

**Table 4:** Median estimates for resistance rates according to country/region (2000–2022)

Countries	Lower limit of mean resistance rate (%)	Upper limit of mean resistance rate (%)	Median estimate (%)
Europe (*n* = 3)	14.1	21.7	14.1
*High Income Countries (HICs)*			
United Kingdom (*n* = 9)	0	74.1	13.2
Ireland (*n* = 1)	N/A	N/A	2.4
USA (*n* = 4)	2.2	60	2.7
Australia (*n* = 1)	N/A	N/A	21
China* (*n* = 2)	8	51	29.5
Japan* (*n* = 2)	12	19	15.5
Greece (*n* = 1)	N/A	N/A	2.4
Austria (*n* = 1)	N/A	N/A	14.3
Switzerland (*n* = 1)	N/A	N/A	18
Spain (*n* = 1)	N/A	N/A	4.8
Italy* (*n* = 2)	25	26	25.5
Korea (*n* = 1)	N/A	N/A	33.3
Oman (*n* = 1)	N/A	N/A	37
*Upper Middle-Income Countries (UMICs)*			
Bosnia and Herzegovina (*n* = 1)	N/A	N/A	0.6
Brazil (*n* = 2)	7.9	9.4	8.65
Iran (*n* = 2)	34	69.8	51.9
*Lower Middle-Income Countries (LMICs)*			
India* (*n* = 2)	5	24.8	14.9
Nigeria* (*n* = 2)	30.8	69.6	50.2
Ethiopia (*n* = 5)	12.5	68.5	29.8

*Mean estimates calculated for countries with 2 papers (even number)

The majority of the publications (*n* = 27; 61.4%) on drug resistance to chloramphenicol and other antibiotics came from developed nations, which could be attributed to higher research output and frequency of drug resistance monitoring activities, compared to developing nations (*n* = 14; 31.8%). A fraction (*n* = 3; 6.8%) of the reviewed studies were regional cohort studies in Europe, which mostly involved the developed European countries, but no country-specific drug resistance rates were given. Among the developing countries, high resistance rates were observed in Iran (69.8%), Nigeria (69.6%) and Ethiopia (68.5%), while the developed countries with high resistance rates were the United Kingdom (74.1%), United States of America (60%) and China (51%). This is in line with similar results from some other publications, which depicted higher antibiotic resistance rates in developing countries than in developed ones due to the overuse of over-the-counter antibiotics-like chloramphenicol that are affordable and easily accessible [5, 8]. Overall, the mean resistance rates to chloramphenicol varied between 0% and 74.1%, with the majority of the studies (86.4%) showing chloramphenicol resistance rates below 50%, and more than half (23 out of 44) of the studies showed resistance rates lower than 20%.. This depicts that high resistance to chloramphenicol was not common among the reviewed studies.

### Discussion

The drug resistance markers in the ophthalmic bacterial pathogens identified from the review were present on three genetic elements—plasmids, transposons, and chromosomes. In terms of the resistance mechanisms, the first and most prevalent drug resistance mechanism to chloramphenicol is the enzymatic inactivation of the drug by acetylation via chloramphenicol acetyltransferases (CATs) [56]. These enzymes are genetically encoded on the *cat* gene and are borne on mobile genetic elements, hence, their role in plasmid-mediated antibiotic resistance [57]. Three types of CATs are known to exist, which include type A, B and C. The type A CATs play important roles in drug resistance against Chloramphenicol and Fusidic acid, while type B CATs, also called xenobiotic acetyltransferases, are known to mediate resistance to chloramphenicol and Streptogramin. Type C CATs are also capable of chloramphenicol acetylation, but their roles in antibiotic resistance are understudied and largely unknown [57]. Many other chloramphenicol resistance mechanisms also exist, such as efflux pump systems, permeability barriers, target site mutations, and inactivation by phosphotransferases [6, 58].

In terms of the drug resistance rates, the linear trend line is indicative of a higher mean resistance rate of ophthalmic bacterial infections to chloramphenicol in developing countries; however, this is inconclusive due to the lack of adequate studies in these areas. This suggests a requirement for more research in the context of developing nations. Differences in the bacterial resistance to chloramphenicol in ophthalmic infections over the period of 2000–2022 could be attributed to variations in the resistance patterns of the causative bacterial strains, geographical differences (countries and region) of the studies, and factors, such as frequency of antimicrobial resistance monitoring and research output. Most of the bacterial pathogens implicated in ophthalmic infections showed a good level of susceptibility (over 60%) to chloramphenicol, except in the case of *Pseudomonas *spp. (*P. aeruginosa* and *P. putida*), which were intrinsically resistant to chloramphenicol and had low susceptibility rates in all the reviewed publications. Our work had some limitations, such as the fact that the reviewed publications were pooled from PubMed and Google Scholar only, leaving out other databases, such as Web of Science, Scopus, Embase, etc., which may also feature publications that might have provided additional insight concerning chloramphenicol resistance. In addition, as much as our review involved studies from various countries and continents globally, the drug resistance rates observed were only study-specific and were not definitive figures representing the actual summation of chloramphenicol resistance in each country.

Overall, the results from the reviewed literature depict that chloramphenicol is still quite active as a topical antibiotic against ophthalmic bacterial infections, as most of the bacterial strains had decent levels of susceptibility to chloramphenicol from their antimicrobial susceptibility profiles. This is similar to various publications that hold the same view [16, 34, 40, 56, 59]. As much as chloramphenicol susceptibility rates were high in the majority of the publications, concerns about increasing chloramphenicol resistance have been raised, such as the study by Lee et al., where increasing drug resistance against chloramphenicol was observed across primary, secondary and tertiary healthcare settings in London, United Kingdom [49]. Some publications hold a view contrary to ours, of chloramphenicol being unsuitable as an empirical eye drop treatment due to increasing rates of drug resistance from frequent prescription and overuse [29, 35, 52]. In light of the observed resistance rates, recommendations were made by Ogbolu et al. and Adebayo et al. for the replacement of topical antibiotics-like chloramphenicol with the third-generation fluoroquinolones (moxifloxacin, gatifloxacin and levofloxacin), due to their improved spectrum of activity and efficacy, as well as the slow rate of emergence of drug resistance due to the double-step mutation required for resistance to develop against these antibiotics [29, 60, 61]. In addition to the above calls for limiting the use of chloramphenicol, the results from a placebo-controlled clinical trial showed that most children with cases of acute infective conjunctivitis were capable of recovering by themselves without the use of chloramphenicol eye drops for therapy [62]. This depicts the need for a reduction in the unnecessary use of antibiotics-like chloramphenicol for relief in mild ophthalmic infections and common ocular irritations.

We recommend enhanced antimicrobial resistance surveillance for the early detection of chloramphenicol-resistant bacterial strains circulating locally in a region or health facility, with the goal of administering more effective second-line therapies once treatment failures are observed. It is also pertinent for antimicrobial guidelines to be regularly reviewed in accordance with local and international drug resistance patterns, for the approval and use of the most effective antibiotics as first-line therapy. In addition, empirical prescription of systemic and ophthalmic antibiotics must be discouraged, and an evidence-based approach of isolation and susceptibility testing of causative bacterial agents must be employed before proceeding to drug prescription. Regulatory policies by appropriate pharmaceutical and health-related bodies are required to control the availability and rate of consumption of over-the-counter medications, such as chloramphenicol eye drops, to lessen the risk of drug resistance.

## Conclusion

Chloramphenicol still appears to be a suitable topical antibiotic for the treatment of ophthalmic bacterial infections; however, rising concerns remain about the drug becoming unsuitable in the long run due to growing rates of drug resistance. Efforts in research and antimicrobial resistance surveillance for chloramphenicol (and other ophthalmic antibiotics) should be intensified in developing countries, as higher rates of chloramphenicol resistance appear to occur in such regions. Overall, antimicrobial stewardship remains vital in combating the trend of drug resistance.

## Data Availability

Not applicable.

## Abbreviations

USA: United States of America
UK: United Kingdom
EMA: European Medicines Agency
MHRA: Medicines and Healthcare Products Regulatory Agency
ASTs: Antimicrobial Susceptibility Tests
CATs: Chloramphenicol Acetyltransferases
